# Impact of rate-pressure product correction of myocardial blood flow on the prognostic relevance in patients with heart failure

**DOI:** 10.1093/ehjci/jeag193

**Published:** 2026-07-24

**Authors:** Thomas Anderton, Bradley Chambers, Maryum Farooq, Chin Yit Soo, Mehak Asad, Michelle Gibbs, Peter Harrison, Osama Tariq, Wasim Javed, Erica Dall'Armellina, Peter Kellman, Sven Plein, Peter P Swoboda, Masafumi Takafuji

**Affiliations:** Leeds Institute of Cardiovascular and Metabolic Medicine, University of Leeds, Leeds, LS2 9JT, United Kingdom; Leeds Institute of Cardiovascular and Metabolic Medicine, University of Leeds, Leeds, LS2 9JT, United Kingdom; Leeds Institute of Cardiovascular and Metabolic Medicine, University of Leeds, Leeds, LS2 9JT, United Kingdom; Leeds Institute of Cardiovascular and Metabolic Medicine, University of Leeds, Leeds, LS2 9JT, United Kingdom; Leeds Institute of Cardiovascular and Metabolic Medicine, University of Leeds, Leeds, LS2 9JT, United Kingdom; Leeds Institute of Cardiovascular and Metabolic Medicine, University of Leeds, Leeds, LS2 9JT, United Kingdom; Leeds Institute of Cardiovascular and Metabolic Medicine, University of Leeds, Leeds, LS2 9JT, United Kingdom; Leeds Institute of Cardiovascular and Metabolic Medicine, University of Leeds, Leeds, LS2 9JT, United Kingdom; Leeds Institute of Cardiovascular and Metabolic Medicine, University of Leeds, Leeds, LS2 9JT, United Kingdom; Leeds Institute of Cardiovascular and Metabolic Medicine, University of Leeds, Leeds, LS2 9JT, United Kingdom; National Institutes for Health, National Heart, Lung, and Blood Institute, Bethesda, MD, USA; Leeds Institute of Cardiovascular and Metabolic Medicine, University of Leeds, Leeds, LS2 9JT, United Kingdom; Leeds Institute of Cardiovascular and Metabolic Medicine, University of Leeds, Leeds, LS2 9JT, United Kingdom; Leeds Institute of Cardiovascular and Metabolic Medicine, University of Leeds, Leeds, LS2 9JT, United Kingdom; Department of Radiology, Mie University Hospital, Tsu, Japan

**Keywords:** rate-pressure product, myocardial blood flow, myocardial perfusion reserve, prognosis, heart failure

## Abstract

**Aims:**

Quantitative perfusion cardiac magnetic resonance (QP-CMR) allows estimation of myocardial blood flow (MBF) and myocardial perfusion reserve (MPR). Resting MBF values are often corrected for the rate-pressure product (RPP). We aimed to assess the effect of RPP correction on MBF values and the prognostic value of QP-CMR in patients with heart failure (HF).

**Methods and results:**

QP-CMR data from 641 prospectively recruited patients with HF and 184 healthy volunteers were analysed. Rest MBF was corrected for RPP (MBFcorr = rest MBFuncorr×10 000/RPP). Corrected MPR was defined as uncorrected stress MBF/rest MBFcorr. The primary endpoint was a composite of heart failure hospitalization (HFH) and all-cause mortality. RPP correction increased rest MBF and decreased MPR to a greater extent in healthy volunteers than in patients. During median follow-up of 3.5 years, 101 events occurred (55 deaths and 46 HFH). Patients who reached the primary endpoint had significantly higher rest MBFuncorr and lower MPRuncorr. Rest MBFuncorr (adjusted hazard ratio per 1 mL/g/min increase, 3.97, 95% confidence interval [CI] 1.93–8.16, *P* < 0.001) and MPRuncorr (adjusted hazard ratio per 1-unit increase, 0.74, 95% CI 0.57–0.95, *P* = 0.024) were significantly associated with the primary endpoint in univariate and multivariate models. After RPP correction, these associations were no longer significant. Kaplan–Meier analysis revealed significant differences in event-free survival for rest MBFuncorr (*P* = 0.005), MPRuncorr (*P* < 0.001), and MPRcorr (*P* = 0.044), but not for rest MBFcorr (*P* = 0.076).

**Conclusion:**

Uncorrected rest MBF and MPR by QP-CMR are independently predictive of adverse outcomes in patients with HF. RPP correction changes MBF and MPR values and attenuates their predictive value.

## Graphical Abstract

**Graphical Abstract:**
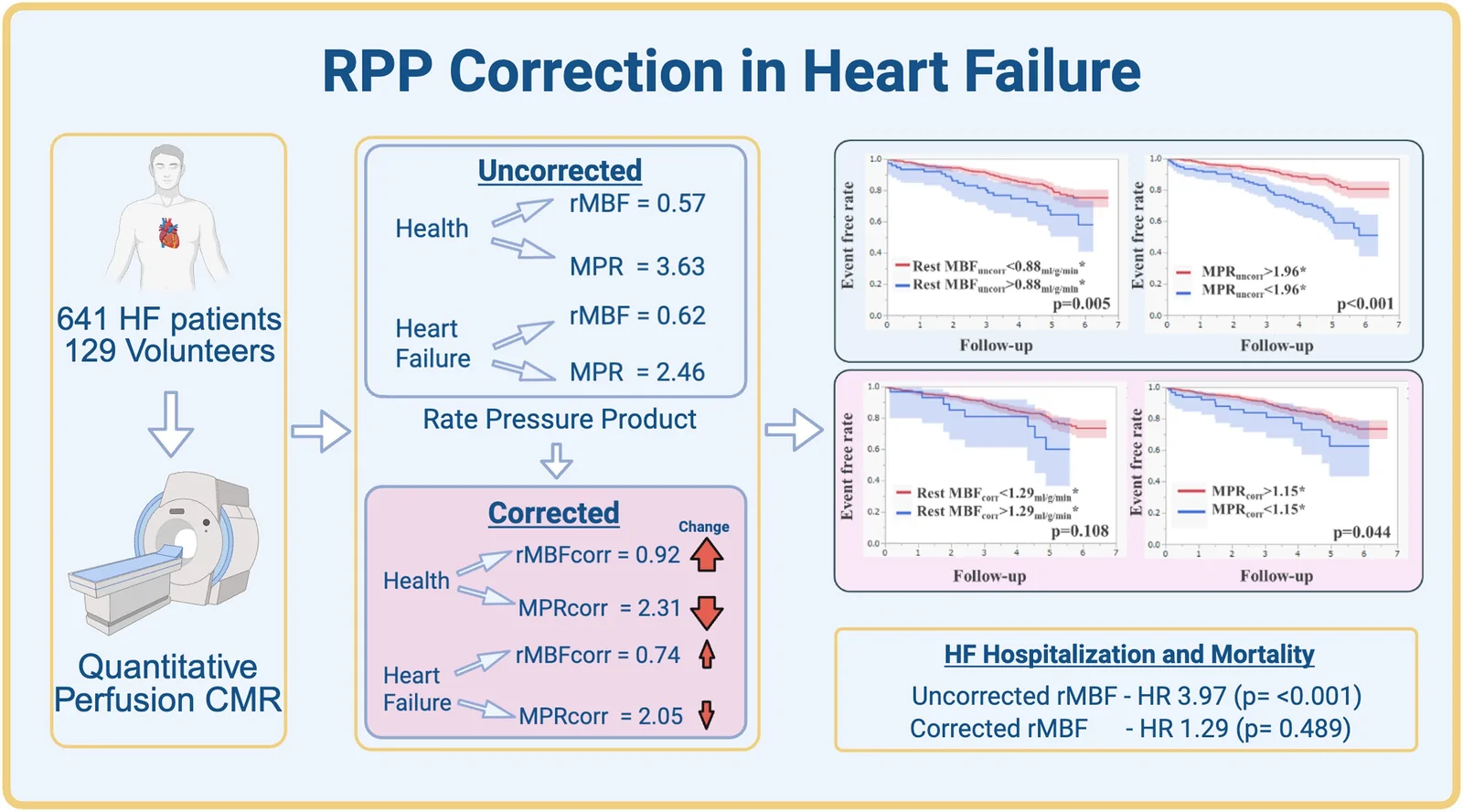

**See the editorial comment for this article ‘On the relevance of resting myocardial blood flow’, by G. Pons-Lladó, https://doi.org/10.1093/ehjci/jeag213.**

## Introduction

Quantitative perfusion (QP) cardiac magnetic resonance (CMR) is an alternative to positron emission tomography (PET) for the estimation of absolute stress and rest myocardial blood flow (MBF) and myocardial perfusion reserve (MPR). Recent guidelines attribute QP-CMR, a Class IIb indication, in patients with angina/ischaemia with non-obstructive coronary arteries.^1^ It has shown utility in multi-vessel coronary artery disease (CAD)^2^ and offers additive prognostic information over visual analysis.^3^ A recent consensus document by the Society for Cardiovascular Magnetic Resonance has provided guidance on the acquisition and analysis of QP-CMR data,^4^ but has not commented on the use of rate-pressure product (RPP) correction of MBF values, which is recommended in international guidelines for QP-PET.^5^

The RPP is defined as a patient’s heart rate (HR, bpm) multiplied by their systolic blood pressure (SBP, mmHg) and can be applied to rest MBF to provide a rest MBF and MPR ‘corrected’ for underlying physiological parameters such as myocardial oxygen demand and coronary driving pressure.^6^ The same is not true of stress MBF, in which coronary driving pressure becomes largely independent of these other physiological factors. RPP is a long-standing clinical marker that has been shown to independently predict major adverse cardiovascular events (MACE) in HF^7^ and CAD^8^ cohorts.

The argument in favour of RPP correction in QP is that an abnormally elevated rest MBF, due to underlying physiological factors rather than inherent cardiac changes, may result in a low MPR despite the presence of normal peak stress MBF and potentially therefore lead to a false-positive diagnosis.^9^ Countering this is the argument that uncorrected values may be capturing the complex mechanisms underlying abnormal coronary flow and therefore offer potential additional prognostic value.^6^

To date, no systematic evaluation of the impact of RPP correction on MBF and MPR values as well as the prognostic value of QP-CMR has been undertaken. We therefore sought to determine the relative influence of RPP on QP-CMR values in both healthy volunteers and HF patients and, subsequently, the effect of RPP correction on the prognostic information offered by QP-CMR in HF.

## Methods

### Study population

We retrospectively analysed QP data derived from patients prospectively recruited into a regional registry of new-onset HF patients. This registry comprises a cohort of patients who were clinically diagnosed with HF at cardiology clinics and referred for CMR between November 2017 and April 2024 (ethical approval 17/YH/0300 and 20/NW/0326).^10,11^ Exclusion criteria were lack of recorded HR and systemic BP during perfusion CMR, presence of structural heart disease (e.g. hypertrophic cardiomyopathy, cardiac sarcoidosis, cardiac amyloidosis), congenital heart disease, and significantly impaired renal function (glomerular filtration rate <30 mL/min/1.73 m^2^). Data on symptoms, comorbidities, and medication use were collected prospectively, and clinical outcomes were collected through electronic health records.

We additionally analysed a departmental group of healthy volunteers, who had no history of cardiac disease, no cardiovascular symptoms, were on no cardiovascular medication, and had no major risk factors for cardiovascular disease. Volunteers were also excluded if their CMR scan showed incidental infarction or significant non-ischaemic fibrosis defined as mid-wall or subepicardial LGE greater than 5 g. All volunteers also underwent blood testing and were excluded if they were found to have NT-proBNP >125 pg/mL.

### CMR acquisition

All CMR studies were conducted on a 3T Magnetom Prisma (Siemens Healthcare, Erlangen, Germany). The study protocol included (i) cine imaging, (ii) adenosine stress and rest perfusion, and (iii) motion-corrected bright-blood LGE with phase-sensitive inversion-recovery sequences in both long- and short-axis orientation (see Supplementary data online, *Appendix S1*).

Stress perfusion imaging was performed during adenosine infusion at a dose of 140 µg/kg/min for a minimum of 3 min using a single-bolus dual sequence that acquired three short-axis slices at every RR interval.^12^ In case of inadequate haemodynamic response (HR increase of <10 bpm) or absence of symptomatic response, the infusion rate was increased progressively up to 210 mcg/kg/min^3^. Rest perfusion images were acquired after a 20 min interval. HR and BP were taken immediately before both rest and stress imaging. The RPP was calculated as pre-rest HR (bpm) × pre-rest SBP (mmHg).

### Image analysis

Image processing and analysis used cvi42 software (Circle Cardiovascular Imaging, Calgary, Alberta, Canada). Left ventricular (LV) volumes and mass were obtained from cine-CMR and indexed to body surface area.^10,11^ Ischaemic scar was defined as subendocardial enhancement with a coronary distribution. All other fibrosis patterns were classified as non-ischaemic, except for fibrosis at the right ventricular insertion points of the interventricular septum, which was not reported. Quantitative analysis of myocardial perfusion was performed automatically by the Gadgetron hardware.^12^ All perfusion maps were systematically quality controlled, including review of arterial input function, R–R interval and respiratory rate, as per the latest international guidelines.^4^ Stress was considered adequate if there was an appropriate HR response (>10 bpm), the occurrence of typical symptoms and/or a clear splenic switch-off on perfusion imaging.^13^ For the primary analysis, stress and rest MBF were calculated as the mean MBF of the mid-ventricular slice segments. In addition, mean MBF values for all three myocardial slices were generated. Segments with myocardial infarction were excluded. MBF values were corrected for RPP using the formula: corrected MBF (MBF_corr_) = uncorrected MBF (MBF_uncorr_) × 10 000/RPP. As a supplemental analysis, rest MBF was also corrected using a scaling constant of 7500: MBF_corr_ = MBF_uncorr_ × 7500/RPP. Uncorrected MPR (MPR_uncorr_) was calculated as the ratio of stress MBF to rest MBF_uncorr_. Corrected MPR (MPR_corr_) was defined as stress MBF divided by rest MBF_corr_.

### Follow-up

Patients were followed up by review of electronic clinical records. The primary outcome was defined as a composite endpoint of heart failure hospitalization (HFH) or all-cause mortality, based on the timing of the first event. The two secondary outcomes included the individual occurrence of HFH or all-cause mortality. Time to outcome was defined from the date of the CMR study until the occurrence of a primary outcome or the censorship at the end of the follow-up period. HFH was defined as admission requiring treatment with intravenous diuretics and/or intensification of medical therapy.

### Statistical analysis

Continuous variables were presented as the median with interquartile range (IQR) and were evaluated using the Mann–Whitney *U* test, Steel–Dwass test, or Kruskal–Wallis test, as appropriate. Categorical variables are expressed as frequency (percentage). Pearson’s correlation coefficient and univariate and multivariate linear regression analyses were conducted to examine how RPP relates to MBF under stress and rest condition. Correlations were categorized by *r*-value as previously described.^14^ All variables with a value of *P* < 0.05 in univariate linear regression analysis were included in a subsequent multivariable linear regression analysis, and when RPP, HR, and SBP remained significant variables, only RPP was retained in the model to avoid multicollinearity. Patients were categorized into two groups based on the presence or absence of the composite endpoint. Univariate and multivariate Cox regression analysis was used to estimate the hazard ratios and confidence intervals (CI) for each QP-CMR parameter in relation to each endpoint based on time to first event and was subsequently adjusted for age, sex, left ventricular ejection fraction (LVEF), presence of inducible ischaemia, and ischaemic LGE simultaneously. To evaluate the change in prognostic value of MBF and MPR associated with RPP correction, net reclassification improvement (NRI) and integrated discrimination improvement (IDI) were calculated. In addition, receiver operating characteristic (ROC) curve analysis was performed, and the area under the ROC curve was compared using the DeLong test. Kaplan–Meier curves were used to estimate event-free rates for the primary endpoints, HFH, or death in all patients, and differences between time-to-event curves were compared using the log-rank test. The cut-off values for the perfusion parameters were defined as the fifth percentile of the values in healthy volunteers or derived from ROC curve analysis using the Youden index. Two-sided *P*-values <0.05 were considered statistically significant. All analyses were performed using the JMP data analysis software (JMP Version 14.2.0, SAS Institute Inc., NC, USA).

### Results

From a total of 702 HF patients and 209 healthy volunteers, 56 HF patients were excluded due to lack of documented HR or SBP, 5 due to cardiac amyloidosis (*n* = 2), cardiac sarcoidosis (*n* = 2), and hypertrophic cardiomyopathy(*n* = 1), leaving 641 HF patients for analysis. Twelve healthy volunteers without documented HR or SBP, four subjects with NT-proBNP >125 pg/mL, one with incidental infarction, and eight incidental significant fibrosis were excluded from the healthy volunteer cohort, leaving a total of 184 healthy volunteers for analysis (*Figure 1*).

**Figure 1:**
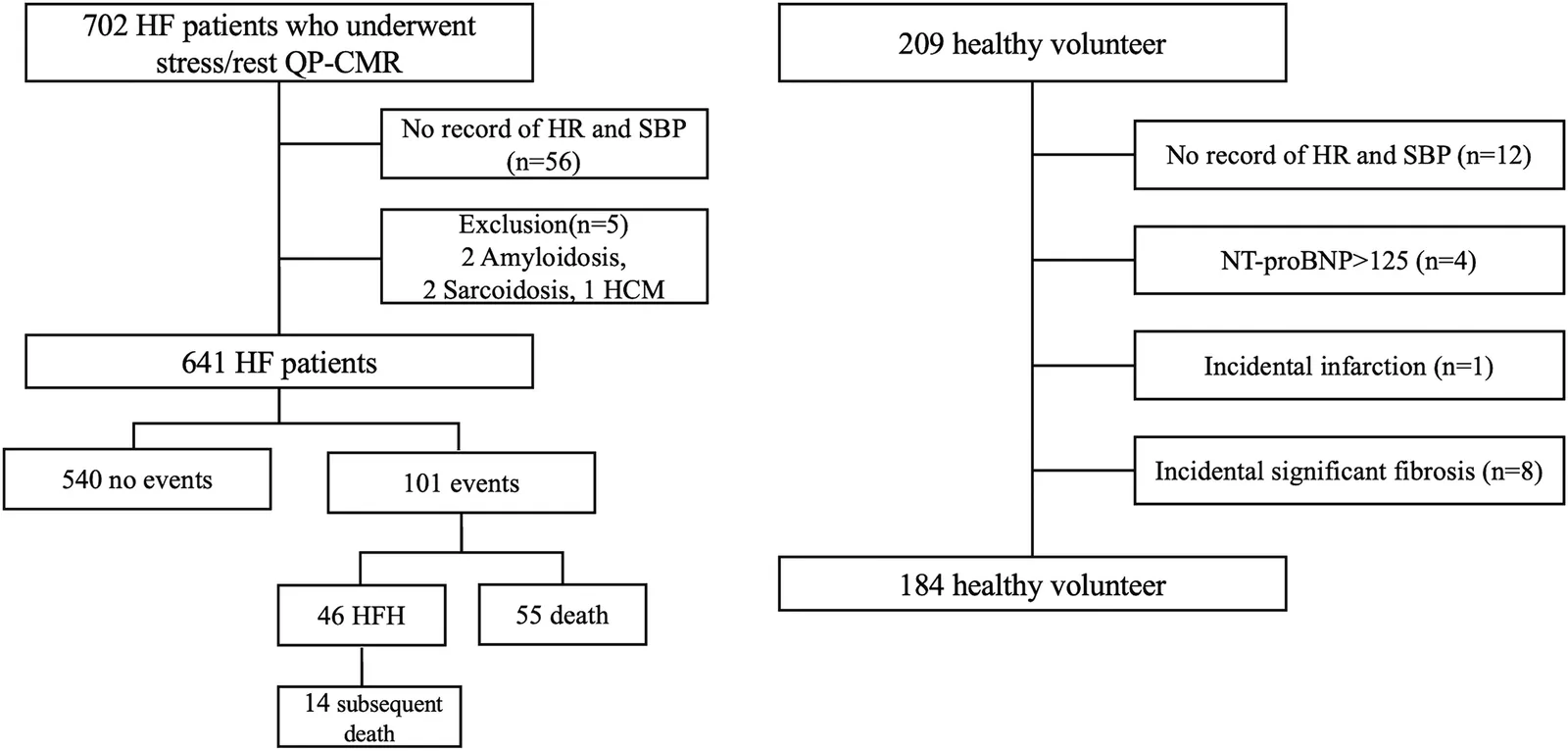
Study flow diagram. HCM, hypertrophic cardiomyopathy; HF, heart failure; HFH, HF hospitalization; HR, heart rate; QP-CMR, quantitative perfusion cardiovascular magnetic resonance; SBP, systolic blood pressure.

Complete follow-up data were available for all 641 HF patients, over a median period of 3.5 [1.8–5.1] years. The primary endpoint occurred in 101 (17.8%) patients (first event; 46 HFH, 55 deaths) at a median of 3.4 [1.8–5.0] years. Fourteen patients with HFH subsequently died after HFH giving a total of 69 deaths (*Figure 1*). Baseline characteristics of healthy volunteers and HF patients in relation to the primary outcome are shown in *Table 1*.

**Table 1:** Baseline characteristics of the patients in relation to the primary outcome

Variable median (interquartile range) or *n* (%)	All HF patients (*n* = 641)	HF patients with event (*n* = 101)	HF patients without event (*n* = 540)	Healthy volunteers (*n* = 184)	*P*-value
**Patient background**					
Age (years)	63 (55–72)	70 (61–76)	63 (55–71)	58 (53–63)	<0.001^a^
Male	410 (64.0)	67 (66.3)	343 (63.5)	108 (58.7)	0.370
BMI (kg/m^2^)	28.4 (24.7–32.2)	28.1 (24.7–31.2)	28.4 (24.7–32.4)	24.2 (22.5–25.9)	<0.001^a^
Hypertension	297 (46.3)	51 (50.5)	246 (45.6)	—	0.361
Diabetes	109 (17.0)	27 (26.7)	82 (15.2)	—	0.005
Hypercholesterolaemia	194 (30.3)	26 (25.7)	168 (31.1)	—	0.281
Current smoking	114 (17.8)	21 (20.8)	93 (17.2)	—	0.389
Past smoking	238 (37.1)	42 (41.6)	196 (36.3)	—	0.313
NYHA class ≥2	304 (47.4)	63 (62.4)	241 (44.6)	—	0.001
Beta-blocker	526 (82.1)	88 (88.0)	438 (81.4)	—	0.112
ACEi/ARB/ARNI	560 (87.4)	89 (88.1)	471 (87.2)	—	0.684
MR	253 (39.5)	33 (33.0)	220 (40.9)	—	0.139
Diuretic	273 (42.6)	52 (52.0)	221 (41.1)	—	0.043
SGLT2i	95 (14.8)	9 (9.0)	86 (16.0)	—	0.072
**CMR parameters**					
LV EDVI (mL/m^2^)	102 (85–127)	104 (89–141)	102 (85–124)	106 (94–117)	0.120^a^
LV ESVI (mL/m^2^)	60 (44–87)	66 (43–97)	59 (44–85)	44 (37–51)	<0.001^a^
LVEF (%)	41 (30–49)	37 (26–49)	42 (31–49)	58 (54–61)	<0.001^a^
LV massI (g/m^2^)	65 (54–79)	70 (57–85)	64 (53–78)	65 (57–72)	0.046^a^
Inducible ischemia	45 (7.0)	9 (8.9)	36 (6.7)	—	0.418
Ischaemic LGE	121 (18.9)	31 (30.7)	90 (16.7)	—	0.001
Non-ischaemic LGE	190 (29.6)	36 (35.6)	154 (28.5)	—	0.150
**HF categories by LVEF**					0.070
HFrEF	307 (47.9)	57 (56.4)	250 (46.3)	—	
HFmrEF	193 (30.1)	21 (20.8)	172 (31.9)	—	
HFpEF	141 (22.0)	23 (22.8)	118 (21.9)	—	

^a^ *P-*values were determined using the Kruskal–Wallis test for comparisons among HF with event, HF without event, and HV, or using the Mann–Whitney *U* test for comparisons between HF with event and HF without event.

ACEi, angiotensin converting enzyme inhibitor; ARB, angiotensin receptor blocker; ARNI, angiotensin receptor-neprilysin inhibitor, BMI, body mass index; MRA, mineralocorticoid receptor antagonist; NYHA, New York Heart Association; SGLT2i, sodium-glucose cotransporter 2 inhibitor.

### QP-CMR and haemodynamic parameters in HF patients and healthy volunteers

QP-CMR and haemodynamic parameters are presented in *Table 2*. Patients who reached the primary endpoint exhibited significantly higher rest RPP and HR than patients who did not reach the primary endpoint and healthy volunteers (*P* < 0.01 for each). In contrast, stress RPP and HR were significantly higher in HF patients compared with healthy volunteers (*P* < 0.01 for each); however, no significant differences of stress RPP and HR were observed between HF patients with and without reaching the primary endpoint (*P* > 0.05).

**Table 2:** CMR characteristics of the patients in relation to the primary outcome

Variable median (IQR)	All HF patients	HF patients with event	HF patients without event	Healthy volunteers	*P*		
					Kruskal–Wallis test	HF with/without events	HF vs. HV
Rest MBF_uncorr_, mL/g/min	0.62 (0.52–0.76)	0.70 (0.58–0.87)	0.61 (0.52–0.73)	0.57 (0.48–0.68)	<0.001	<0.001	<0.001
Rest MBF_corr_, mL/g/min	0.74 (0.62–0.93)	0.77 (0.64–0.97)	0.73 (0.61–0.92)	0.92 (0.78–1.06)	<0.001	0.276	<0.001
Rest RPP, bpm*mmHg	8357 (7020–9960)	9006 (7245–10952)	8255 (6964–9773)	6336 (5518–7403)	<0.001	0.002	<0.001
Rest HR, bpm	68 (60–79)	73 (63–82)	68 (60–78)	53 (48–59)	<0.001	0.008	<0.001
Rest SBP, mmHg	120 (108–135)	125 (109–141)	119 (108–134)	117 (110–124)	0.020	0.082	0.030
Stress MBF, mL/g/min	1.56 (1.22–1.95)	1.48 (1.12–1.93)	1.57 (1.25–1.95)	2.16 (1.81–2.49)	<0.001	0.160	<0.001
Stress RPP, bpm*mmHg	10062 (8361–12050)	9711 (8345–11512)	10147 (8362–12081)	8570 (7526–10113)	<0.001	0.408	<0.001
Stress HR, bpm	82 (73–93)	81 (71–90)	83 (73–94)	71 (63–80)	<0.001	0.092	<0.001
Stress SBP, mmHg	121 (109–138)	121 (110–140)	120 (108–137)	120 (113–130)	0.720	—	—
MPR_uncorr_	2.46 (1.94–3.11)	2.04 (1.43–2.72)	2.53 (2.03–3.14)	3.63 (3.08–4.40)	<0.001	<0.001	<0.001
MPR_corr_	2.05 (1.57–2.66)	1.93 (1.38–2.42)	2.07 (1.58–2.69)	2.31 (2.00–2.79)	<0.001	0.025	<0.001

HR, heart rate; IQR, inter quantile range; MBF, myocardial blood flow; MPR, myocardial perfusion reserve; MBF_uncorr_, uncorrected myocardial blood flow, MBF_corr_, RPP-corrected myocardial blood flow, MPR_uncorr_, uncorrected myocardial perfusion reserve, MPR_corr_, RPP-corrected myocardial perfusion reserve, RPP, rate pressure product; SBP, systolic blood pressure.

Regarding QP parameters, patients who reached the primary endpoint had significantly higher rest MBF_uncorr_ and lower MPR_uncorr_ in the mid LV slice than both patients who did not reach the primary endpoint and healthy volunteers (*P* < 0.01 each). Rest MBF_corr_ and MPR_corr_ did not differ significantly between HF subgroups, but healthy volunteers had higher rest MBF_corr_ and MPR_corr_ than those with HF with and without events. The results of the global perfusion parameters using all three slices were similar and are summarized in Supplementary data online, *Table S1*. RPP-corrected rest MBF and MPR using 7500 as the constant are summarized in Supplementary data online, *Table S2*.

The cut-off values for QP-CMR parameters, defined as the fifth percentile of the values in healthy volunteers, were as follows: rest MBF_uncorr_, 0.88 mL/g/min; rest MBF_corr_, 1.29 mL/g/min; stress MBF, 1.07 mL/g/min; MPR_uncorr_, 1.96; and MPR_corr_, 1.15.

### Relationship between RPP and MBF in healthy volunteers and HF patients

In healthy volunteers, rest MBF_uncorr_ correlated moderately with rest RPP and rest HR, whereas stress MBF correlated weakly with stress RPP and stress HR. Rest MBF_uncorr_ and stress MBF did not significantly correlate with rest and stress SBP (*Figure 2*).

**Figure 2:**
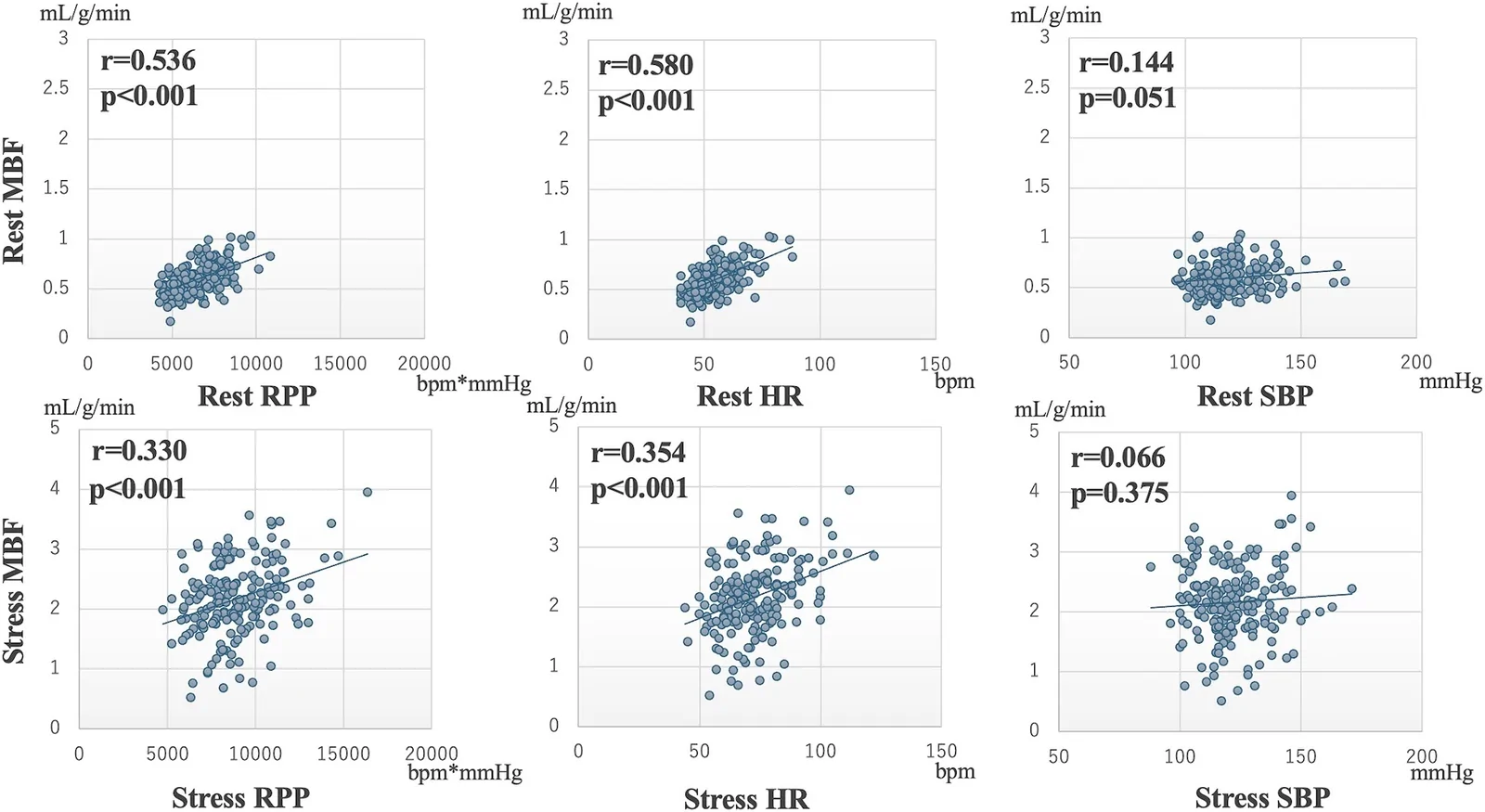
Correlation between MBF and haemodynamic parameters in healthy volunteers.

In univariate linear regression analysis of healthy volunteers, rest MBF_uncorr_ was significantly associated with rest RPP, rest HR, LV end-diastolic volume index (EDVI), LVEF, and LV mass index (see Supplementary data online, *Table S3*), whereas stress MBF was significantly associated only with stress RPP and stress HR (see Supplementary data online, *Table S4*). In multivariate analysis, rest RPP was independently associated with rest MBF_uncorr_, and stress RPP was independently associated with stress MBF.

In HF patients (*Figure 3*), rest MBF_uncorr_ correlated weakly with rest RPP, rest HR, and very weakly with rest SBP, whereas stress MBF correlated very weakly with stress RP and stress HR. Stress MBF did not significantly correlate with stress SBP. The results of correlation between global stress MBF and rest MBF_uncorr_ and haemodynamic parameters in HF patients are demonstrated in Supplementary data online, *Figure S1*.

**Figure 3:**
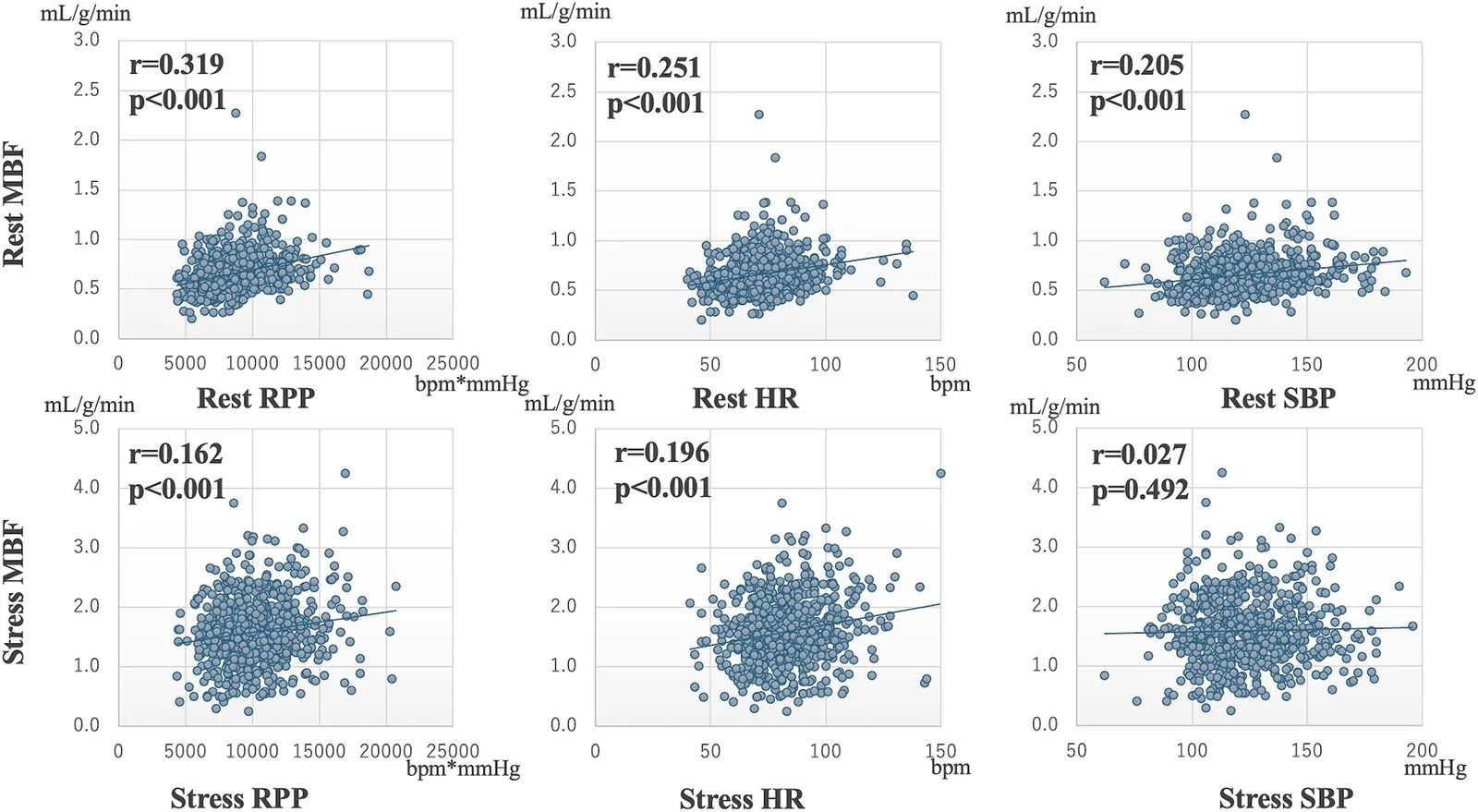
Correlation between MBF and haemodynamic parameters in HF patients.

In univariate linear regression analysis, rest MBF_uncorr_ was significantly associated with rest RPP, rest HR, rest SBP, male sex, body mass index (BMI), current smoking, mineralocorticoid receptor antagonist (MRA), sodium-glucose cotransporter 2 inhibitor (SGLT2i), LV EDVI, LVEF, LV mass index and non-ischaemic LGE (*P* < 0.05 for each). In multivariate analysis including the variables that were significant in the univariate analysis, rest RPP was independently associated with rest MBF_uncorr_ in HF patients (see Supplementary data online, *Table S5*). The results of the univariate and multivariable linear regression analyses of global rest MBF_uncorr_ in patients with HF are summarized in Supplementary data online, *Table S6*.

In univariate linear regression analysis of HF, stress MBF was significantly associated with stress RPP, stress HR, age, male, BMI, hypercholesterolaemia, diabetes, hypertension, past smoking, NYHA class, MRA, diuretic, SGLT2i, statin, LV EDVI, LVEF, LV mass index, inducible ischaemia, and ischaemic and non-ischaemic LGE (*P* < 0.05 for each). In multivariate analysis including the variables that were significant in the univariate analysis, stress RPP was independently associated with stress MBF in HF patients (see Supplementary data online, *Table S7*). The results of the univariate and multivariable linear regression analyses of global stress MBF in patients with heart failure are summarized in Supplementary data online, *Table S8*.

### Impact of RPP correction of MBF on the prognostic value in HF patients

Kaplan–Meier analysis demonstrated significant differences in primary endpoint event-free survival according to the cut-off values in rest MBF_uncorr_ (*P* = 0.005), stress MBF (*P* = 0.005), MPR_uncorr_ (*P* < 0.001), and MPR_corr_ (*P* = 0.044) for prediction of the primary outcome (*Figure 4*). In contrast, stratification according to rest MBF_corr_ showed no significant difference in event-free survival (*P* = 0.108).

**Figure 4:**
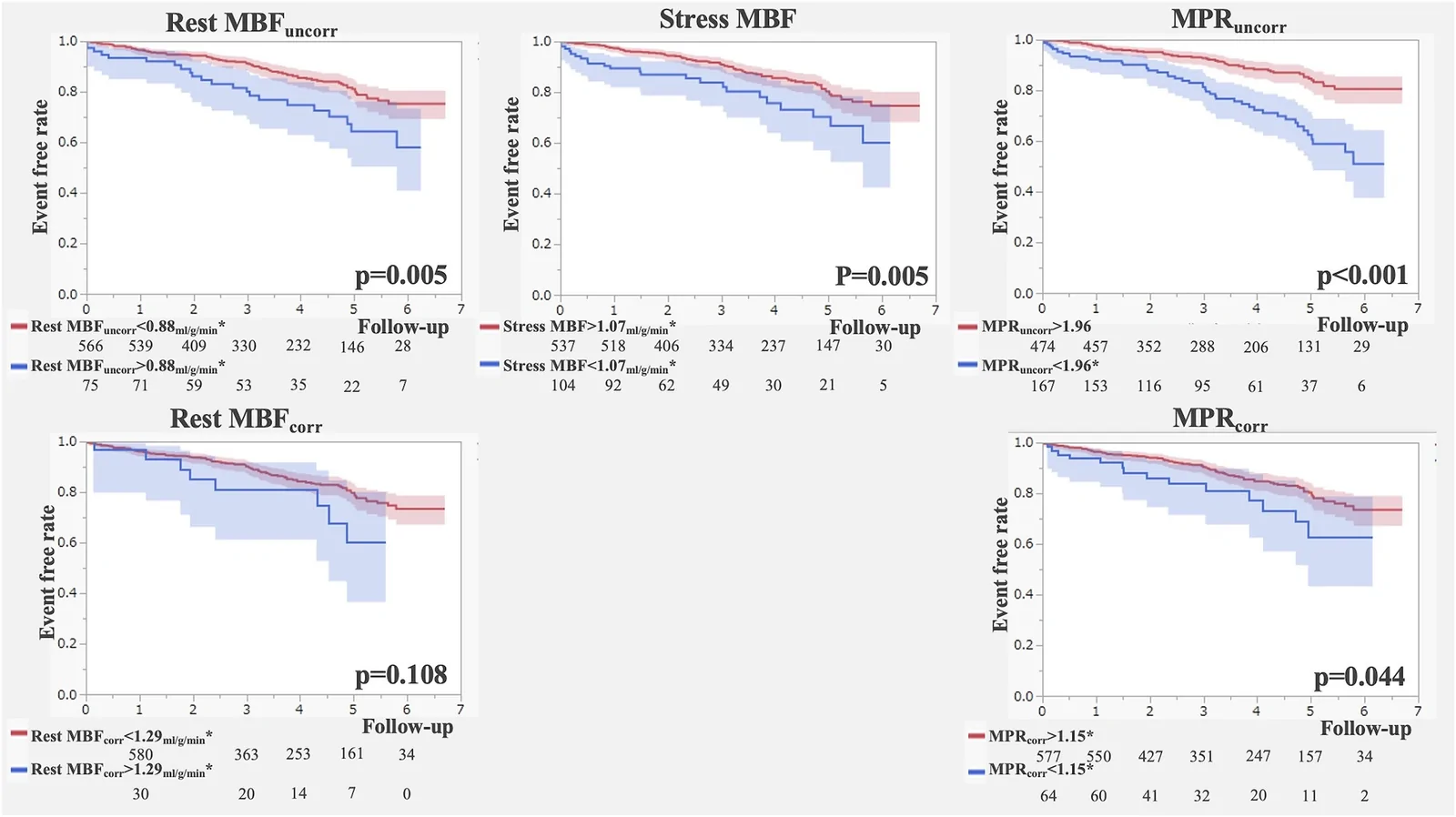
Kaplan–Meier survival curves of rest MBF_uncorr_, rest MBF_corr_, stress MBF, MPR_uncorr_, and MPR_corr_ for the primary outcome.

For the secondary outcomes, MPR_uncorr_ was associated with both mortality (*P* < 0.001) and HFH (*P* < 0.001). Rest MBF_uncorr_ (*P* = 0.004), stress MBF (*P* = 0.002), and MPR_corr_ (*P* = 0.003) were associated with HFH but not with mortality (*P* > 0.05 each). Rest MBF_corr_ was associated with death (*P* = 0.044) but not with HFH (*P* = 0.181) (see Supplementary data online, *Figures S2* and *S3*).

Cox regression analysis for each parameter in relation to the primary endpoint is shown in *Table 3*. Rest MBF_uncorr_ (adjusted hazard ratio = 3.97, *P* < 0.001) and MPR_uncorr_ (adjusted hazard ratio = 0.74, *P* = 0.024) were associated with the occurrence of the primary outcome on univariate and multivariate analysis. However, after RPP correction, these associations were no longer statistically significant (rest MBF_corr_, adjusted hazard ratio = 1.29, *P* = 0.489; MPR_corr_, adjusted hazard ratio = 0.84, *P* = 0.179). While stress MBF was associated with the occurrence of the primary outcome in univariate analysis, this association was not significant after adjustment in multivariable analysis (adjusted hazard ratio = 0.90, *P* = 0.605).

**Table 3:** Cox regression for perfusion parameters and the primary endpoint

	Primary outcome: composite of death or HFH			
Predictor	Unadjusted hazard ratio (95% CI)	*P-*value	Adjusted hazard ratio^a^ (95% CI)	*P-*value
Rest MBF_uncorr_	3.43 (1.52–6.84)	0.001	3.97 (1.93–8.16)	<0.001
Rest MBF_corr_	1.13 (0.53–2.31)	0.749	1.29 (0.63–2.62)	0.489
Stress MBF	0.68 (0.47–0.99)	0.045	0.90 (0.60–1.33)	0.605
MPR_uncorr_	0.65 (0.51–0.84)	0.001	0.74 (0.57–0.95)	0.024
MPR_corr_	0.75 (0.58–0.96)	0.028	0.84 (0.65–1.07)	0.179

^a^Model adjusted for age, sex, LVEF, and presence of inducible ischaemia and ischaemic LGE.

CI, confidence interval; HFH, heart failure hospitalization.

RPP correction significantly reduced the continuous NRI and IDI for the predictive value of both MPR (NRI, −0.291, *P* = 0.007; IDI, −0.017, *P* < 0.001, respectively) and rest MBF (NRI, −0.426, *P* < 0.001; IDI, −0.030, *P* < 0.001, respectively).

The results of Kaplan–Meier survival curves and Cox regression analysis for global perfusion parameters are demonstrated in Supplementary data online, *Figure S4* and Supplementary data online, *Table S9*.

Cox regression analyses of rest MBF and MPR corrected using a scaling constant of 7500 for the primary endpoint are summarized in Supplementary data online, *Table S10*.

The AUCs for rest MBF_uncorr_ and MPR_uncorr_ were 0.620 (95% CI, 0.558–0.683) and 0.638 (95% CI, 0.575–0.701), respectively. After RPP correction, the AUCs significantly decreased to 0.534 (95% CI, 0.471–0.597) for rest MBF_uncorr_ (*P* = 0.003) and 0.570 (95% CI, 0.510–0.630) for MPR_uncorr_ (*P* = 0.002) (see Supplementary data online, *Figure S5*). Kaplan–Meier curve analysis stratified using cut-off values derived from the ROC curves showed similar results with uncorrected rest MBF and MPR significantly predictive of outcomes but not RPP- corrected rest MBF (see Supplementary data online, *Figure S6*).

## Discussion

It remains uncertain whether QP-CMR data should be corrected for RPP and current practice is inconsistent. This study is the first to systematically investigate the impact of RPP correction on QP-CMR data and to assess the prognostic relevance of RPP adjustment on QP CMR results in a large, prospectively recruited cohort of patients. Overall, the findings of this study add weight to the argument against the use of RPP correction of MBF and MPR in QP-CMR.

### Influence of RPP on MBF

In healthy volunteers, RPP correction resulted in a near doubling of resting MBF. This led to a significant reduction in MPR from 3.61 to 2.31, which is much lower than expected for a healthy cohort and close to commonly used thresholds of abnormality. RPP correction in HF patients had a lesser effect on rest MBF and MPR than in healthy volunteers, largely due to their higher resting HR, and consequently, RPP correction narrowed the differences between healthy volunteers and patients. These results suggest that RPP correction results in unphysiologically low MPR estimates and impairs the distinction between health and disease.

There was significant positive correlation between HR, BP, and RPP, and MBF, though the findings were not consistent between healthy and HF participants. Except for BP and rest MBF, the correlation between HR, BP, and RPP with MBF was notably stronger in healthy participants than in HF. Multivariate analysis confirmed the significance of the relationship between RPP and MBF at both rest and stress in HF, but again with a smaller apparent overall effect than multivariate analysis in healthy volunteers. This suggests that in health the RPP may be a greater factor in determining MBF, whereas in HF, the complex regulatory mechanisms of the myocardial circulation, in addition to the non-cardiac factors less present in health, become of greater relevance. To ‘correct’ to RPP without also attempting to correct for other relevant factors—for example, haematocrit, age, and gender—does not appear to be physiologically valid.^15^

### Prognostic effect of RPP correction

Correction of QP-CMR data to RPP reduced the prognostic value of the data in our cohort of HF patients. While uncorrected MPR as well as uncorrected rest MBF were strong predictors of the primary combined endpoint as well as the secondary endpoints of HFH and death, corrected MPR was a much weaker predictor of both the primary endpoint and HFH, whilst no longer being predictive of all-cause mortality alone. Corrected rest MBF was not predictive of either the primary or secondary endpoints. Particularly notable was the marked reduction of hazard ratio of combined HFH and mortality from 3.97 to 1.29 when using corrected rest MBF.

This observation likely relates to the reduced difference in rest MBF and MPR between groups with RPP correction. As would be expected, those with HF and events were older, had higher NYHA class, and were more likely to be diabetic with ischaemic LGE than HF patients without events. Compared to patients without events and healthy volunteers, HF patient with events had higher HR, BP, RPP, and uncorrected rest MBF with a lower stress MBF and uncorrected MPR. As noted, the overall effect of correction of rest MBF was to reverse the difference in rest MBF—with healthy volunteers subsequently having significantly higher values—and to negate much of the MPR difference between groups, including between patients with and without events.

Between-group differences in medication use were small, with a slightly higher beta-blocker use in those with events and a higher mineralocorticoid receptor antagonist use in those without events. Small studies have shown that beta-blocker use decreases RPP and resting MBF in PET^16^ without evidence showing the effect of the other HF medication. Despite the higher beta-blocker use, however, in our cohort those with events had higher, not lower HR. The overall effect from these medications on both the RPP and outcomes in participants in this study is difficult to fully adjust for, though could account for differences in outcomes between patients.

### RPP correction in PET

A seminal study by Czernin *et al*^17^ investigated the effect of age, sex and haemodynamic parameters on MBF using ^13^N-Ammonia PET. They concluded that, due to the significant correlation between rest MBF and RPP (*r* = 0.72), the observed decrease in MPR seen in advancing age was primarily due to haemodynamic parameters and not a ‘true’ fall in MPR. Thereafter, RPP correction has been used almost ubiquitously in PET-QP studies, but has most recently been questioned and investigated.

Two recent retrospective analyses investigated the prognostic implications of RPP correction in PET QP. Huck *et al*^18^ retrospectively evaluated >3000 patients clinically referred for PET and evaluated the effect of RPP correction on MACE prediction over a median 7-year follow-up period. RPP correction reclassified around 20% of each group (initially defined as normal MPR >2 or abnormal <2). However, RPP correction did not improve MACE prediction in either situation, with MPR <2 predictive of MACE regardless of the corrected MPR and a 6% MACE rate in discordant MPR_corr_ <2 vs. 15% in discordant MPR_corr_ >2. Similarly, in a large retrospective analysis, Rifai *et al*^19^ found MPR <2 to be predictive of MACE even if corrected MPR was >2. Unlike our work, these studies were retrospectively performed in an undifferentiated cohort clinically referred for scans. They do, however, suggest the impact of RPP correction is not a modality-specific finding but rather a more generalizable one.

### RPP in the CMR literature

As in PET, there are no prospective studies specifically investigating the relevance of RPP on the diagnostic or prognostic capability of QP-CMR. Perhaps due to this lack of evidence, the use of RPP correction in CMR is inconsistent, with several studies utilizing it,^20,21^ whilst others reporting uncorrected values.^22–24^ The most common justification for correction is the correlation between RPP and rest MBF, alongside the prior noted study by Czernin *et al*,^17^ employing PET, not CMR.

Brown *et al.*^25^ noted the correlation of RPP with rest MBF in the currently largest study of normal QP values in CMR. Whilst the significant effect of RPP correction was noted by the authors, no advocacy is made to definitively use corrected values, and it was noted that the significant sex-based differences in rest MBF persisted even after RPP correction, potentially confirming the significance of other factors. Similarly in test-retest variability work by the same group,^26^ RPP and, in particular, HR correction was employed to attempt to reduce the variability of rest MBF in repeat testing. However, doing so in this cohort did not fully remove the differences in rest MBF seen.

### Limitations

The primary analysis in this study was limited to the mid ventricular slice. With the pulse sequence used, data for this slice are acquired in systole, which minimizes partial volume effects and provides the highest accuracy in QP-CMR. We also provide data for all three slices, which showed similar findings, albeit with less discrimination between groups. There were differences in the medication prescription between our heart failure groups. This has the possible dual effect of altering the resting MBF alongside prognostic implications. The generalizability of these findings to non-heart failure patients, for example, those with CAD, is unclear and warrants investigation. This work also does not directly inform on the relevance of RPP correction when using QP for diagnostic purposes. A proportion of this cohort of healthy volunteers were veteran athletes, leading to them having relatively high LV-EDVi. The threshold for stress MBF in this study was relatively low, which is likely a result of using below the fifth centile as the cut-off for stress MBF and using a post-processing model that produces MBF values at the lower end of the published literature.

## Conclusion

Uncorrected rest MBF and MPR by QP-CMR are independently predictive of adverse outcomes in patients with HF. RPP correction changes MBF and MPR values and attenuates their predictive value in HF patients. These findings suggest that RPP correction for QP-CMR masks important physiological and prognostic information and should not be used in clinical routine. Future studies will be required to determine the effect of RPP correction on the diagnostic accuracy of QP-CMR.

## Supplementary Material

jeag193_Supplementary_Data

## Data Availability

The data underlying this article will be shared on reasonable request to the corresponding author.
