# Supplementary material for: Impact of rate-pressure product correction of myocardial blood flow on the prognostic relevance in patients with heart failure
Source: Eur Heart J Cardiovasc Imaging. 2026 Jul 24;27(10):1817–26. doi: 10.1093/ehjci/jeag193 (PMC13623416; doi:10.1093/ehjci/jeag193)
Supplement: Supplementary file 1 [file jeag193_supplementary_data.zip › FINAL Supplemental materials and appendices.docx]

**Supplemental materials and appendices**

**Supplemental Figure 1: Correlation between global MBF and hemodynamic parameters in HF patients**

MBF, myocardial blood flow; SBP, systolic blood pressure; HR, heart rate; RPP, rate pressure product.

**Supplemental Figure 2:** Kaplan–Meier survival curves of rest MBF_uncorr_, rest MBF_corr_, stress MBF, MPR_uncorr_, and MPR_corr_ for heart failure hospitalization.

MBF_uncorr_, uncorrected myocardial blood flow, MBF_corr_, RPP-corrected myocardial blood flow, MPR _uncorr_, uncorrected myocardial perfusion reserve, MPR _corr_, RPP-corrected myocardial perfusion reserve.

**Supplemental Figure 3:** Kaplan–Meier survival curves of rest MBF_uncorr_, rest MBF_corr_, stress MBF, MPR_uncorr_, and MPR_corr_ for all-cause mortality.

MBF_uncorr_, uncorrected myocardial blood flow, MBF_corr_, RPP-corrected myocardial blood flow, MPR _uncorr_, uncorrected myocardial perfusion reserve, MPR _corr_, RPP-corrected myocardial perfusion reserve.

**Supplemental Figure 4:** Kaplan–Meier survival curves for the primary endpoint according to global values of rest MBF_uncorr_, rest MBF_corr_, stress MBF, MPR_uncorr_, and MPR_corr_.

MBF_uncorr_, uncorrected myocardial blood flow, MBF_corr_, RPP-corrected myocardial blood flow, MPR _uncorr_, uncorrected myocardial perfusion reserve, MPR _corr_, RPP-corrected myocardial perfusion reserve.

**Supplemental Figure 5:** ROC curve analysis of rest MBF_uncorr_, rest MBF_corr_, stress MBF, MPR_uncorr_, and MPR_cor_ for predicting the primary outcome.

AUC area under the curve, CI, confidence interval. Other abbreviations as **Supplemental Figure 2**

**Supplemental Figure 6:** Kaplan–Meier survival curves for the primary endpoint stratified by ROC-derived cutoff values for rest MBF_uncorr_, rest MBF_corr_, stress MBF, MPR_uncorr_, and MPR_corr_.

Abbreviations as **Supplemental Figure 2**

| **Variable**  **median (IQR)** | **All HF patients** | **HF patients**  **with event** | **HF patients**  **without event** | **p**** |
| --- | --- | --- | --- | --- |
| Rest MBF_uncorr_, mL/g/min | 0.62 (0.52–0.73) | 0.67 (0.53–0.85) | 0.61 (0.51–0.73) | 0.005 |
| Rest MBF_corr_, mL/g/min | 0.74 (0.60–0.91) | 0.76 (0.58–0.95) | 0.73 (0.60–0.91) | 0.698 |
| Stress MBF, mL/g/min | 1.60 (1.25–2.00) | 1.49 (1.21–1.91) | 1.61 (1.28–2.04) | 0.058 |
| MPR_uncorr_ | 2.57 (1.99–3.25) | 2.20 (1.66–2.76) | 2.62 (2.06–3.32) | <0.001 |
| MPR_corr_ | 2.11 (1.59–2.74) | 1.97 (1.49–2.56) | 2.15 (1.61–2.81) | 0.056 |

**Supplemental Table 1: Global perfusion parameters of the patients in relation to the primary outcome**

*p-values are from Kruskal–Wallis test. HR, heart rate; IQR, inter quantile range; MBF, myocardial blood flow; MPR, myocardial perfusion reserve; RPP, rate pressure product; SBP, systolic blood pressure.

**Supplemental Table 2: RPP-Corrected Resting MBF and MPR Using 7500 as the Constant**

| **Variable**  **median (IQR)** | **All HF patients** | **HF patients**  **with event** | **HF patients**  **without event** | **Healthy volunteers** | **p value** | | |
| --- | --- | --- | --- | --- | --- | --- | --- |
|  |  |  |  |  | **Kruskal–Wallis test*** | **HF with/without events** | **HF vs HV** |
| Rest MBF_corr_, mL/g/min | 0.55 (0.46–0.70) | 0.58 (0.48–0.73) | 0.55 (0.46–0.69) | 0.69 (0.58–0.80) | <0.001 | 0.276 | <0.001 |
| MPR_corr_ | 2.73 (2.10–3.55) | 2.57 (1.85–3.23) | 2.76 (2.11–3.58) | 3.09 (2.66–3.71) | <0.001 | 0.025 | <0.001 |

Abbreviations as Table 2.

**Supplemental Table 3. Univariate and Multivariate linear regression analysis of rest MBF**_uncorr_ **in heathy volunteers.**

|  | **Univariate analysis** | | | **Multivariate analysis** | |
| --- | --- | --- | --- | --- | --- |
|  | **B** | **95%CI** | **p** | **Standardized β** | **p** |
| **Patient background** | | | | | |
| Age | -0.001 | -0.004–0.001 | 0.317 |  |  |
| Male | -0.024 | -0.068–-0.020 | 0.282 |  |  |
| BMI | 0.002 | -0.007–-0.010 | 0.700 |  |  |
| Rest RPP | 0.00006 | 0.00005–0.00008 | <0.001 | 0.514 | <0.001 |
| Rest HR | 0.010 | 0.008–0.012 | <0.001 |  |  |
| Rest SBP | 0.002 | -0.000003–0.003 | 0.051 |  |  |
| **CMR parameters** | | | | | |
| LV EDVI (mL/m^2^) | -0.003 | -0.004–-0.001 | <0.001 | 0.165 | 0.078 |
| LVEF (%) | 0.007 | 0.003–0.011 | <0.001 | 0.162 | 0.011 |
| LV mass index (g/m^2^) | -0.004 | -0.006–-0.002 | <0.001 | -0.273 | 0.003 |

BMI, body mass index; BP, blood pressure; CI, confidence intervals; EDVI, end-systolic volume index; EF ejection fraction; HR, heart rate; LV, left ventricular; RPP, rate pressure product; SBP, systolic blood pressure.

**Supplemental Table 4. Univariate and Multivariate linear regression analysis of stress MBF**_uncorr_ **in healthy volunteers**

|  | **Univariate analysis** | | | **Multivariate analysis** | |
| --- | --- | --- | --- | --- | --- |
|  | **B** | **95%CI** | **p** | **Standardized β** | **p** |
| **Patient background** | | | | | |
| Age | -0.003 | -0.013–0.007 | 0.566 |  |  |
| Male | -0.170 | -0.346–0.006 | 0.059 |  |  |
| BMI | 0.028 | -0.004–0.061 | 0.088 |  |  |
| Stress RPP | 0.00010 | 0.00006–0.00014 | <0.001 | 0.330 | <0.001 |
| Stress HR | 0.016 | 0.010–0.022 | <0.001 |  |  |
| Stress SBP | 0.003 | -0.003–0.009 | 0.375 |  |  |
| **CMR parameters** | | | | | |
| LV EDVI (mL/m^2^) | -0.003 | -0.008–0.002 | 0.245 |  |  |
| LVEF (%) | 0.003 | -0.014–0.019 | 0.762 |  |  |
| LV mass index (g/m^2^) | -0.006 | -0.014–0.001 | 0.090 |  |  |

BMI, body mass index; CI, confidence intervals; EDVI, end-systolic volume index; EF ejection fraction; HR, heart rate; LV, left ventricular; RPP, rate pressure product; SBP, systolic blood pressure.

**Supplemental Table 5. Univariate and Multivariate linear regression analysis of rest MBF**_uncorr_ **in patients with heart failure**

|  | **Univariate analysis** | | | **Multivariate analysis** | |
| --- | --- | --- | --- | --- | --- |
|  | **B** | **95%CI** | **p** | **Standardized β** | **p** |
| **Patient background** | | | | | |
| Age | -0.0004 | -0.0017–-0.0008 | 0.507 |  |  |
| Male | -0.105 | -0.136–-0.074 | <0.001 | -0.124 | <0.001 |
| BMI | -0.007 | -0.010–-0.004 | <0.001 | -0.151 | <0.001 |
| Hypercholesterolemia | -0.006 | -0.040–0.028 | 0.742 |  |  |
| Diabetes | 0.004 | -0.037–0.045 | 0.853 |  |  |
| Hypertension | -0.008 | -0.039–0.023 | 0.625 |  |  |
| Current smoking | 0.075 | 0.035–0.115 | <0.001 | 0.128 | <0.001 |
| Past smoking | -0.027 | -0.059–0.005 | 0.099 |  |  |
| NYHA class | 0.014 | -0.010–0.038 | 0.240 |  |  |
| Betablocker | -0.036 | -0.077–0.005 | 0.086 |  |  |
| ACEi/ARB/ARNI | -0.073 | -0.091–0.002 | 0.064 |  |  |
| MRA | -0.086 | -0.118–-0.055 | <0.001 | -0.056 | 0.127 |
| Diuretic | -0.030 | -0.062–0.001 | 0.058 |  |  |
| SGLT2i | -0.131 | -0.174–-0.088 | <0.001 | -0.142 | <0.001 |
| Statin | 0.014 | -0.018–0.045 | 0.395 |  |  |
| Rest RPP | 0.00003 | 0.00002–0.00003 | <0.001 | 0.308 | <0.001 |
| Rest HR | 0.003 | 0.002–0.004 | <0.001 |  |  |
| Rest SBP | 0.002 | 0.001–0.003 | <0.001 |  |  |
| **CMR parameters** | | | | | |
| LV EDVI (mL/m^2^) | -0.0004 | -0.0009–-0.00005 | 0.041 | 0.155 | 0.010 |
| LVEF (%) | 0.003 | 0.002–0.004 | <0.001 | 0.170 | <0.001 |
| LV mass index (g/m^2^) | -0.001 | -0.002–-0.0004 | 0.003 | -0.156 | 0.005 |
| Inducible ischemia | 0.023 | -0.038–0.084 | 0.466 |  |  |
| Ischemic LGE | 0.004 | -0.036–0.043 | 0.860 |  |  |
| Non-ischemic LGE | -0.078 | -0.112–-0.045 | <0.001 | -0.082 | 0.023 |

ACEi, angiotensin converting enzyme inhibitor; ARB, angiotensin receptor blocker; ARNI, angiotensin receptor-neprilysin inhibitor, BMI, body mass index; BP, blood pressure; CI, confidence intervals; EDVI, end-systolic volume index; EF ejection fraction; HR, heart rate; LGE, late gadolinium enhancement; LV, left ventricular; MRA, mineralocorticoid receptor antagonist; NYHA, New York Heart Association; RPP, rate pressure product; SBP, systolic blood pressure; SGLT2i, sodium-glucose cotransporter 2 inhibitor.

**Supplemental Table 6: Univariate and Multivariate linear regression analysis of global rest MBF**_uncorr_ **in patients with heart failure**

|  | **Univariate analysis** | | | **Multivariate analysis** | |
| --- | --- | --- | --- | --- | --- |
|  | **B** | **95%CI** | **p** | **Standardized β** | **p** |
| **Patient background** | | | | | |
| Age | -0.0009 | -0.0022–0.0004 | 0.181 |  |  |
| Male | -0.101 | -0.133–-0.069 | <0.001 | -0.135 | <0.001 |
| BMI | -0.005 | -0.008–-0.002 | 0.002 | -0.091 | 0.017 |
| Hypercholesterolemia | 0.0007 | -0.034–0.035 | 0.967 |  |  |
| Diabetes | -0.018 | -0.060–0.025 | 0.414 |  |  |
| Hypertension | -0.020 | -0.052–0.012 | 0.215 |  |  |
| Current smoking | 0.065 | 0.023–0.106 | 0.002 | 0.107 | 0.007 |
| Past smoking | -0.039 | -0.071–-0.006 | 0.022 | -0.025 | 0.514 |
| NYHA class | 0.005 | -0.020–0.030 | 0.688 |  |  |
| Betablocker | -0.011 | -0.053–0.031 | 0.602 |  |  |
| ACEi/ARB/ARNI | -0.047 | -0.095–0.001 | 0.056 |  |  |
| MRA | -0.083 | -0.115–-0.050 | <0.001 | -0.076 | 0.050 |
| Diuretic | -0.034 | -0.066–-0.002 | 0.040 | 0.003 | 0.939 |
| SGLT2i | -0.111 | -0.155–-0.066 | <0.001 | -0.116 | 0.002 |
| Statin | 0.006 | -0.027–0.038 | 0.730 |  |  |
| Rest RPP | 0.00002 | 0.00002–0.00003 | <0.001 | 0.227 | <0.001 |
| Rest HR | 0.003 | 0.002–0.004 | <0.001 |  |  |
| Rest SBP | 0.002 | 0.001–0.002 | <0.001 |  |  |
| **CMR parameters** | | | | | |
| LV EDVI (mL/m^2^) | -0.0005 | -0.0009–-0.00005 | 0.027 | 0.083 | 0.194 |
| LVEF (%) | 0.002 | 0.001–0.003 | <0.001 | 0.079 | 0.102 |
| LV mass index (g/m^2^) | -0.001 | -0.002–-0.0004 | 0.002 | -0.126 | 0.030 |
| Inducible ischemia | -0.011 | -0.074–0.051 | 0.725 |  |  |
| Ischemic LGE | -0.007 | -0.048–0.034 | 0.742 |  |  |
| Non-ischemic LGE | -0.078 | -0.112–-0.043 | <0.001 | 0.090 | 0.019 |

ACEi, angiotensin converting enzyme inhibitor; ARB, angiotensin receptor blocker; ARNI, angiotensin receptor-neprilysin inhibitor, BMI, body mass index; BP, blood pressure; CI, confidence intervals; EDVI, end-systolic volume index; EF ejection fraction; HR, heart rate; LGE, late gadolinium enhancement; LV, left ventricular; MRA, mineralocorticoid receptor antagonist; NYHA, New York Heart Association; RPP, rate pressure product; SBP, systolic blood pressure; SGLT2i, sodium-glucose cotransporter 2 inhibitor.

**Table 7. Univariate and Multivariate linear regression analysis of stress MBF**_uncorr_ **in patients with heart failure**

|  | **Univariate analysis** | | | **Multivariate analysis** | |
| --- | --- | --- | --- | --- | --- |
|  | **B** | **95%CI** | **p** | **Standardized β** | **p** |
| **Patient background** | | | | | |
| Age | -0.010 | -0.014–-0.007 | <0.001 | -0.120 | 0.004 |
| Male | -0.249 | -0.338–-0.159 | <0.001 | -0.112 | 0.005 |
| BMI | -0.011 | -0.019–-0.002 | 0.011 | -0.074 | 0.053 |
| Hypercholesterolemia | -0.132 | -0.228–-0.037 | 0.007 | -0.067 | 0.122 |
| Diabetes | -0.213 | -0.330–-0.097 | <0.001 | -0.056 | 0.155 |
| Hypertension | -0.221 | -0.307–-0.134 | <0.001 | -0.105 | 0.010 |
| Current smoking | -0.005 | -0.120–0.110 | 0.934 |  |  |
| Past smoking | -0.178 | -0.268–-0.088 | <0.001 | -0.106 | 0.004 |
| NYHA | -0.080 | -0.148–-0.012 | 0.021 | -0.047 | 0.203 |
| Betablocker | -0.090 | -0.207–0.026 | 0.128 |  |  |
| ACEi/ARB/ARNI | -0.073 | -0.205–0.060 | 0.283 |  |  |
| MRA | -0.115 | -0.206–-0.025 | 0.012 | 0.002 | 0.949 |
| Diuretic | -0.246 | -0.334–-0.159 | <0.001 | -0.086 | 0.029 |
| SGLT2i | -0.188 | -0.311–-0.064 | 0.003 | -0.008 | 0.837 |
| Statin | -0.113 | -0.202–-0.023 | 0.013 | 0.065 | 0.149 |
| Stress RPP | 0.00003 | 0.0002–0.0005 | <0.001 | 0.129 | <0.001 |
| Stress HR | 0.007 | 0.004–0.010 | <0.001 |  |  |
| Stress SBP | 0.0008 | -0.0014–0.0029 | 0.492 |  |  |
| **CMR parameters** | | | | | |
| LV EDVI (mL/m^2^) | -0.003 | -0.004–-0.002 | <0.001 | 0.084 | 0.184 |
| LVEF (%) | 0.012 | 0.008–0.015 | <0.001 | 0.139 | 0.004 |
| LV mass index (g/m^2^) | -0.007 | -0.009–-0.005 | <0.001 | -0.205 | <0.001 |
| Inducible ischemia | -0.266 | -0.437–-0.094 | 0.002 | -0.049 | 0.220 |
| Ischemic LGE | -0.198 | -0.310–-0.086 | <0.001 | 0.005 | 0.901 |
| Non-ischemic LGE | -0.137 | -0.233–-0.041 | 0.005 | 0.007 | 0.852 |

ACEi, angiotensin converting enzyme inhibitor; ARB, angiotensin receptor blocker; ARNI, angiotensin receptor-neprilysin inhibitor, BMI, body mass index; CI, confidence intervals; EDVI, end-systolic volume index; EF ejection fraction; HR, heart rate; LGE, late gadolinium enhancement; LV, left ventricular; MRA, mineralocorticoid receptor antagonist; NYHA, New York Heart Association; SBP, systolic blood pressure; SGLT2i, sodium-glucose cotransporter 2 inhibitor RPP, rate pressure product.

**Supplemental Table 8: Univariate and Multivariate linear regression analysis of global stress MBF**_uncorr_ **in patients with heart failure**

|  | **Univariate analysis** | | | **Multivariate analysis** | |
| --- | --- | --- | --- | --- | --- |
|  | **B** | **95%CI** | **p** | **Standardized β** | **p** |
| **Patient background** | | | | | |
| Age | -0.011 | -0.014–-0.007 | <0.001 | -0.136 | 0.002 |
| Male | -0.186 | -0.276–-0.095 | <0.001 | -0.079 | 0.054 |
| BMI | -0.012 | -0.020–-0.004 | 0.005 | -0.095 | 0.016 |
| Hypercholesterolemia | -0.083 | -0.179–0.013 | 0.089 |  |  |
| Diabetes | -0.145 | -0.262–-0.028 | 0.015 | -0.018 | 0.652 |
| Hypertension | -0.225 | -0.312–-0.138 | <0.001 | -0.132 | 0.001 |
| Current smoking | 0.033 | -0.082–0.148 | 0.574 |  |  |
| Past smoking | -0.189 | -0.279–-0.099 | <0.001 | -0.116 | 0.002 |
| NYHA | -0.105 | -0.173–-0.037 | 0.002 | -0.081 | 0.033 |
| Betablocker | -0.063 | -0.179–0.054 | 0.291 |  |  |
| ACEi/ARB/ARNI | -0.118 | -0.250–0.015 | 0.081 |  |  |
| MRA | -0.136 | -0.226–-0.046 | 0.003 | -0.025 | 0.532 |
| Diuretic | -0.180 | -0.269–-0.092 | <0.001 | -0.028 | 0.491 |
| SGLT2i | -0.198 | -0.322–-0.075 | 0.002 | -0.027 | 0.493 |
| Statin | -0.122 | -0.211–-0.032 | 0.008 | 0.023 | 0.574 |
| Stress RPP | 0.00003 | 0.00002–0.00005 | <0.001 | 0.108 | 0.007 |
| Stress HR | 0.007 | 0.004–0.010 | <0.001 |  |  |
| Stress SBP | 0.0006 | -0.002–0.003 | 0.592 |  |  |
| **CMR parameters** | | | | | |
| LV EDVI | -0.003 | -0.004–-0.001 | <0.001 | -0.024 | 0.709 |
| LVEF | 0.010 | 0.006–0.013 | <0.001 | 0.086 | 0.082 |
| LV mass index | -0.006 | -0.008–-0.004 | <0.001 | -0.106 | 0.075 |
| Inducible ischemia | -0.183 | -0.355–-0.011 | 0.037 | -0.027 | 0.511 |
| Ischemic LGE | -0.144 | -0.256–-0.032 | 0.012 | 0.017 | 0.695 |
| Non-ischemic LGE | -0.154 | -0.250–-0.058 | 0.002 | -0.028 | 0.488 |

ACEi, angiotensin converting enzyme inhibitor; ARB, angiotensin receptor blocker; ARNI, angiotensin receptor-neprilysin inhibitor, BMI, body mass index; CI, confidence intervals; EDVI, end-systolic volume index; EF ejection fraction; HR, heart rate; LGE, late gadolinium enhancement; LV, left ventricular; MRA, mineralocorticoid receptor antagonist; NYHA, New York Heart Association; SBP, systolic blood pressure; SGLT2i, sodium-glucose cotransporter 2 inhibitor RPP, rate pressure product.

**Supplemental Table 9: Cox regression analysis of global perfusion parameters for the primary endpoint**

|  | **Primary outcome: Composite of Death or HFH** | | | |
| --- | --- | --- | --- | --- |
| **Predictor** | Unadjusted HR (95% CI) | P value | Adjusted HR* (95%CI) | P value |
| **Rest MBF_uncorr_** | 2.39 (1.03–4.93) | 0.029 | 3.04 (1.31–6.24) | 0.005 |
| **Rest MBF_corr_** | 0.99 (0.45–2.05) | 0.988 | 1.14 (0.54–2.23) | 0.710 |
| **Stress MBF** | 0.64 (0.44–0.91) | 0.016 | 0.84 (0.56–1.24) | 0.380 |
| **MPR_uncorr_** | 0.64 (0.50–0.81) | <0.001 | 0.72 (0.57–0.92) | 0.009 |
| **MPR_corr_** | 0.78(0.61–0.99) | 0.045 | 0.87 (0.68–1.09) | 0.241 |

*Model adjusted for age, sex, LVEF and presence of inducible ischemia and ischemic LGE.

Abbreviations as Table 3.

**Supplemental Table 10: Cox regression analysis of rest MBF and MPR corrected using a scaling constant of 7500 for the primary endpoint**

|  | **Primary outcome: Composite of Death or HFH** | | | |
| --- | --- | --- | --- | --- |
| **Predictor** | Unadjusted HR (95% CI) | P value | Adjusted HR* (95%CI) | P value |
| **Rest MBF_corr_** | 0.90 (0.59–1.35) | 0.623 | 1.03 (0.68–1.52) | 0.875 |
| **MPR_corr_** | 0.75 (0.58–0.96) | 0.028 | 0.84 (0.65–1.08) | 0.167 |

*Model adjusted for age, sex, LVEF and presence of inducible ischemia and ischemic LGE.

Abbreviations as Table 3.

**Appendix 1**

CMR for each patient was performed in the following sequence:

1. Scout images to determine LV short axis.
2. LV function (cine) imaging in standard long and short axis planes using a fast gradient echo sequence; 10-12 slices; 30 phases; 10/0mm; to assess regional and global LV function.
3. Stress perfusion imaging; Pharmacological stress was induced via adenosine infusion through a peripheral intravenous cannula at 140 mcg/kg/min for 3 minutes. The rate of infusion was increased to 175 mcg/kg/min after 2 minutes if there was no evidence of hemodynamic or symptomatic response to stress. Patients were monitored throughout adenosine infusion for symptoms with heart rate and blood pressure recorded at 2 minute intervals. Adequate response to adenosine infusion was assessed by the presence of symptoms, hemodynamic response (>10% in heart rate), splenic “switch-off” and visual assessment of quantitative perfusion maps with repeat stress performed in cases of inadequate response. An intravenous bolus of 0.05mmol/kg gadolinium-based contrast agent (Gadovist, Bayer, Leverkusen, Germany) was administered at 5ml/s followed by a 20ml saline flush using an automated injection pump (Medrad MRXperion Injection System, Bayer). Myocardial perfusion maps were acquired using a MOCO free-breathing, dual sequence T1-weighted, saturation recovery prepared gradient echo, in three short-axis 8mm slices at the basal, mid and apical levels with slice spacing varying on a per-patient basis to cover the entire LV; 90 dynamic images per slice. The dual sequence single bolus perfusion sequence has been previously described^22^. Separately optimized sequences were used for myocardium and blood pool. In-line automatic reconstruction and post-processing were implemented within the Gadgetron software framework.
4. Rest perfusion imaging; pulse sequence and geometry identical to stress perfusion imaging described above without administration of adenosine.
5. Top up of non-ionic gadolinium-based contrast agent (further 0.05mmol/kg Gadovist® or 0.1 mmol/kg Dotarem) was given immediately following rest perfusion imaging.
6. MOCO LGE imaging; bright blood LGE images were acquired in matching LV short-axis and long-axis planes 6 minutes after contrast administration with additional dark blood images acquired if required.
